# Goldstandard TUR-P: the bigger the volume, the higher the speed, but resected volumes of more than 60g are critical

**DOI:** 10.1007/s11255-024-04329-6

**Published:** 2024-12-21

**Authors:** Heinrich Schulte-Baukloh, Markus Weber, Dirk Höppner, Holger Heidenreich, Thorsten Schlomm, Sarah Weinberger, Thomas Enzmann, Bernhard Ralla

**Affiliations:** 1 Department of Urology, Charité—University Hospital Berlin, Hindenburgdamm 30, 12203 Berlin, Germany; 2 Urologic Practice Turmstrasse, Berlin-MitteMoabit, Germany; 3 Department of Urology, Bundeswehrkrankenhaus, Berlin, Germany; 4 Department of Urology, University Hospital Brandenburg, Brandenburg, Germany

**Keywords:** Prostate, BPE, Benign prostatic enlargement, Monopolar TUR-P, Resection prostate

## Abstract

**Background:**

According to the European Association of Urology guidelines, the limit for monopolar, transurethral resection (M-TURP) in BPH- therapy is a volume of 80 g. However, whether larger prostates can also be resected transurethrally might also depend on the experience of the surgeon and especially the resected volume and speed of the resection. Little is known about the latter, and this paper aims to these factors.

**Methods:**

This study included 540 patients who received a single-stage M-TURP. Based on the postoperative resection weight, these were divided into four groups: group 1 with 10 to 59.9 g, group 2 with 60 to 79.9 g, group 3 with 80 to 99.9 g, and group 4 with ≥ 100 g. We examined patient age, the ASA-score, the IPSS, quality of life, resection weight, time and speed, pre- and postoperative hemoglobin and serum sodium values, complications, and surgeon experience.

**Results:**

The mean resection weight was 41.6 g, and the mean values for resection time and speed were 61.3 min and 0.7 g/min, respectively. The resection speed increased significantly with resection weight (from 0.7 to 1.3 g/min) as well as with the surgeon’s level of experience (from 0.4 to 0.9 g/min). The number of serious complications (Clavien–Dindo ≥ IIIb) increased significantly from a resection volume of 59.5 g (cut-off value).

**Summary:**

The resection speed of M-TURP increased significantly with the resection weight and the surgeon's level of experience. Regardless of speed, resection weight of more than 60 g might increase the risk of severe complications.

## Introduction and aims

Monopolar transurethral resection of the prostate (M-TURP) is the most frequently used surgical treatment of benign prostatic hyperplasia (BPH) in many countries, although there are meanwhile various competing procedures, especially the bipolar TUR-P (which reduced the risk of sodium losses and TUR-P syndrome due to the use of normal saline), but also other techniques such as different laser resection, holmium laser enucleation, and various vaporization, and embolization techniques [1]. In 2019, 71.7% of the operation procedures for BPH in Germany were performed as M-TUR-P. M-TURP has been developed and optimized continuously over the past decades, and no other surgical technique offers comparable long-term results [2, 3]. Despite increasing numbers of competing methods for de-obstructing the prostate, the monopolar TUR technique is a long-standing sophisticated technology subject to consistent optimization and has been made more user-friendly and safer for the patient. Advances include, for example, a continuous irrigation lock, a double lock system with an all-round rotating inner jacket, or precise electrode guidance through a working element.

The indication for this procedure is failure or progression under drug therapy in patients with lower urinary tract symptoms secondary to benign prostatic obstruction and a prostate size of 30 to a maximum of 80 mL [1], although there is no conclusive evidence for this size limitation. Accordingly, the current EAU guidelines (2024) mention that only a few RCTs have been performed in patients with a prostate volume of > 80 mL [1]. A meta-analysis of 20 M-TURP RCTs with follow-up periods of up to 5 years revealed an improvement in the mean Qmax of + 162%, a reduction in the IPSS score of − 70%, and a reduction in the residual urine volume of − 77% [4]. Technical advances have mitigated the most severe complications and morbidities, which include, e.g., bleeding, TUR syndrome, urinary incontinence, retrograde ejaculation, bladder neck or urethral stenosis, and erectile dysfunction [4–7]. The mortality rate of TURP is about 0.25% [8].

The risk factors for intraoperative complications of bleeding and TUR syndrome are extended surgical time, increased prostate size, and surgeon’s experience/ expertise.

However, limited data are available on how the amount of resection affects the speed of resection; what influence the amount of resection has on the complication rate (not the preoperatively estimated volume); and whether surgeon's expertise can level out the possible risk of larger amounts of resection using a faster speed of resection.

Further, evidence that patients with larger prostate volumes > 80 mL should not be operated on transurethrally is lacking. Thus, we focused on these parameters.

## Patients and methods

This retrospective study (Ethics committee number EA2-154–24, University Hospital Charité, Berlin, Germany) involved clinical data collected from 565 patients who underwent transurethral prostate resection over 4 years. Patients whose resection weight was not specified in the histopathological report, and those with a resection weight of < 10 g were excluded (*n* = 25). Very few patients, those with significant coagulation disorders, were excluded from the analysis.

### Patients

The following data were extracted from the medical records (only the parameters relevant to the study question were listed): age, ASA classification, IPSS score, perioperative Hb loss in grams/deciliter (calculated using the difference from the preoperative Hb level), lowest Hb level (measured within 24 h after surgery), need for blood transfusion, lowest serum sodium level (measured within 24 h after surgery), surgical duration (in minutes), resection speed (in grams/minute), and resected prostate volume determined by the pathologist (in grams). The preoperatively determined sonographic prostate sizes could not be evaluated from the medical records owing to incomplete documentation.

### Classification of complications (Clavien–Dindo)

To present the perioperative complications in a practical manner and close to the everyday urological practice, we divided them according to the Clavien–Dindo [9] classification into (1) none, (2) ≤ grade IIIa complications (such as prolonged gross hematuria requiring irrigation, cystitis, epididymitis, temporary urinary retention in the postoperative course, tamponade without the need for surgical intervention, fever, and pollakiuria after catheter removal) and (3) ≥ grade IIIb complications (such as perforation, tamponade, or bleeding requiring surgical intervention; urosepsis; postoperative stroke; TUR syndrome; intraoperative asystole; or postoperative myocardial infarction).

### Classification of the surgical short-term outcome up to the time of discharge (approximately 5 days)

“0” indicates no improvement.

"1" denotes improvement defined by the following criteria:

• Free micturition postoperatively;

• Increase in the maximum flow of 10 mL/s; and

• Residual urine-free micturition (<100 mL)

"2" indicates deterioration defined by the following criteria:

• Decrease in the maximum flow by more than 10 mL/s;

• Catheter derivation at the time of discharge;

• Residual urine volume of >100 mL.

Although therapeutic success rate was not a primary variable in this study, the parameters mentioned here were highly predictive of at least primary therapeutic success soon after the operation. Postoperative IPSS and PSA values ​​were not recorded due to the short time and volatility of the parameters after the operation. Furthermore, the long-term outcome of M-TURP was not the focus of this study and was thus not evaluated.

### Classification of surgeons’ experience

"1" denotes the highest level of surgical experience (> 300 TURPs): department heads and senior physicians (two surgeons).

"2" denotes a high level of surgical experience (50–299 TURPs): senior physicians (three surgeons).

“3” indicates the lowest level of surgical experience (< 50 TURPs): urologic specialists and residents in training (four surgeons).

This classification refers to the surgeons’ wealth of experience at the end of the evaluation period.

The following devices were used: monopolar Richard Wolf resectoscope (Knittlingen, Germany) as the M-TURP device and ERBE-Elektromedizin (Tübingen, Germany) Vio 300D as the HF device. The surgical technique used was based on the classic TUR-P (according to [10]). Great importance was attached to passing on the exact surgical procedure in a standardized manner, from more experienced surgeons (‘teachers’) to the less experienced (‘students’), to achieve the highest possible consistency of good surgical results: using a resectoscope, a resection trench was formed at 6 o'clock in the lithotomy position up to the surgical capsule, while protecting the colliculus seminalis. Subsequently, the lateral lobes at 3 and 9 o'clock and the ventral parts up to the capsule were resected. The apical resection is important for the success of the therapy: the resectoscope was aligned with the colliculus and the apical resection was carried out in a semicircular manner from 6 to 12 o'clock with meticulous protection of the external sphincter. Enucleation techniques were not used. There was no general patient selection, but resections with a preoperatively estimated resection weight greater than 40 g were generally performed by experienced or very experienced surgeons.

### Statistical analysis

For categorical data, absolute and relative frequencies (based on non-missing data) were calculated. For continuous data, means, standard deviations, medians, and minimum and maximum values were calculated. This involved showing the distribution of age, IPSS score, ASA classification, resected prostate volume, surgical duration, surgical speed, Hb loss, sodium level, complications, and outcome of the entire patient group.

After a pre-analysis of the rate of severe complications ≥ IIIb in relation to a resection weight of up to 80 g, we calculated the cut-off values for the occurrence of severe complications based on a receiver-operating characteristic (ROC) curve analysis.

Thereafter, we divided the patients into four groups (according to the ROC curve analysis): group 1 with a resection weight of 10–59.9 g; group 2, 60–79.9 g; group 3, 80–99.9 g; and group 4, ≥ 100 g. Descriptive statistics were generated for age, ASA classification, and IPSS score. The surgical speed and Hb loss were compared using the Kruskal–Wallis test, followed by a paired analysis. The postoperative sodium level, complications, outcome, and administration of erythrocyte concentrates were evaluated using Fisher's exact test.

For calculation of the differences regarding surgeon’s experience, we divided the total sample population into three groups: group 1, operated on by a very experienced surgeon; group 2, an experienced surgeon; and group 3, a less-experienced surgeon. The resection speed and Hb loss were compared between these groups using the Kruskal–Wallis test and the postoperative sodium level and outcome using Fisher’s exact test. The three groups divided according to surgeon’s experience were compared using analysis of variance (ANOVA) with subsequent pairwise comparisons. All relevant dependent variables were tested for normal distribution (Shapiro–Wilk test). Small deviations from normality were accepted, since no non-parametric test for interaction effects was available. For pairwise comparisons, type I error was controlled by dividing the overall alpha error (5%) by the number of pairwise comparisons.

## Results

***Age, ASA classification, and IPSS score:*** The mean patient age at surgery was 70.7 (range = 48–89, median = 71) years. In 14 of the 565 patients, no ASA classification could be obtained from the patient files. The patients were grouped according to the ASA classification by the anesthesiologist as follows: ASA I, 24 patients (4.2%); ASA II, 356 patients (63.0%); ASA III, 169 patients (29.9%); ASA IV, 2 patients (0.4%). The mean sum of the IPSS/ QoL scores was 24.2 (median = 25).

**Fig. 1:**
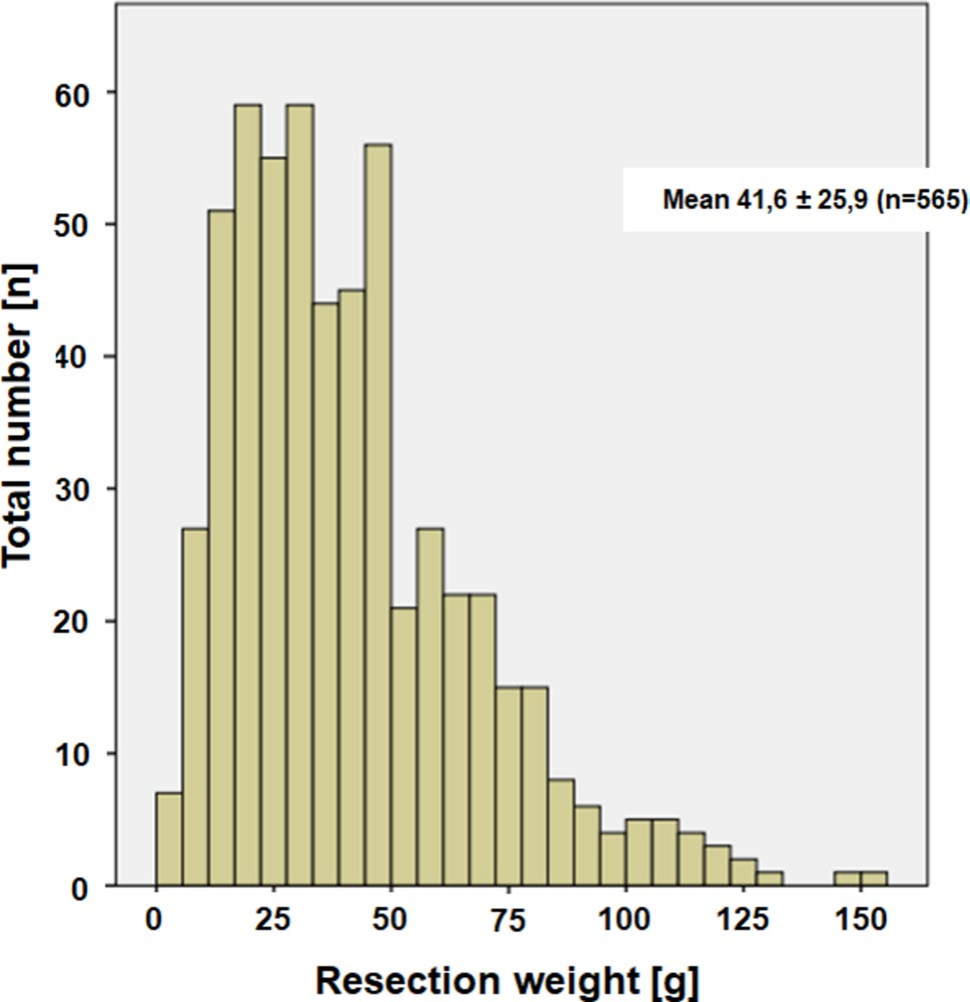
Distribution of the resection weight in grams of the total patient sample

**Total resection weight, speed, complication, and outcome:** The mean resection weight was 41.6 (maximum = 152.7, median = 36) g (Fig. 1).

**Fig. 2:**
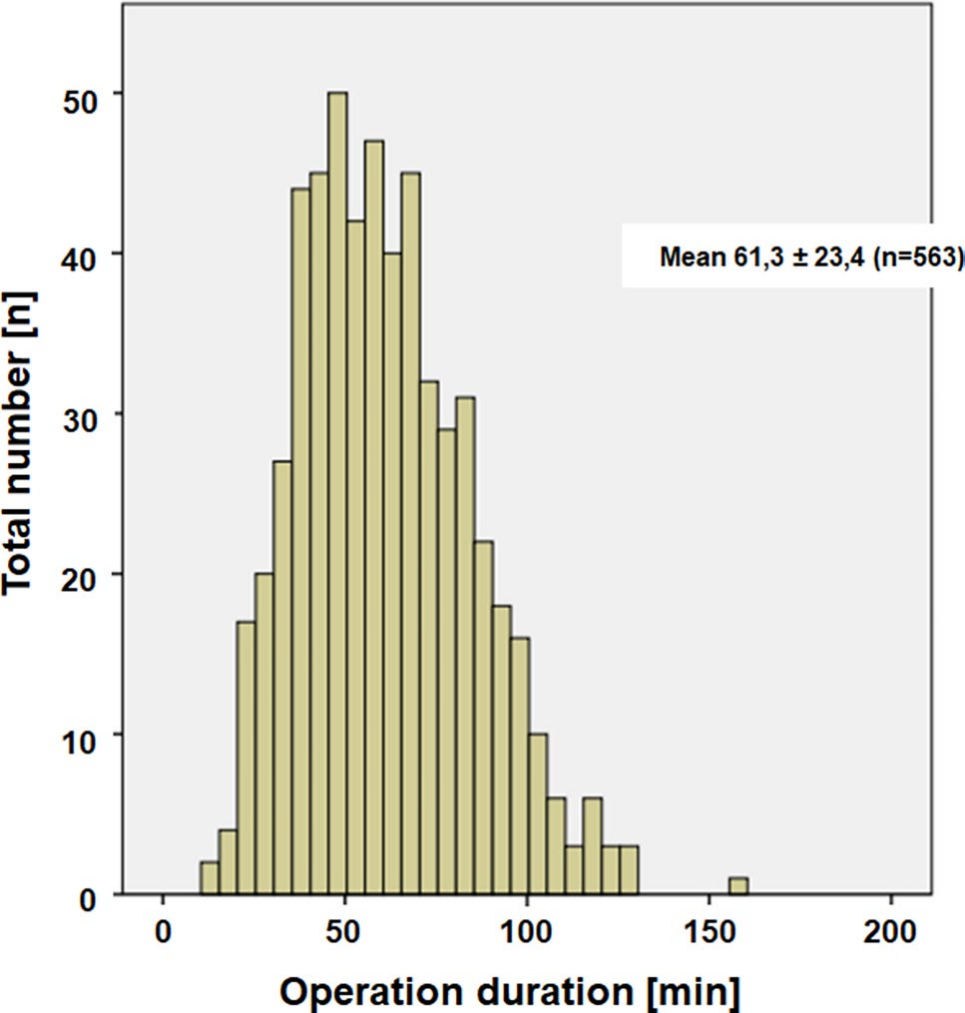
Distribution of the duration of the operation for the entire patient sample

The mean surgical time in 563 patients was 61.3 (maximum = 159, median = 58) min (Fig. 2).

Among all 565 patients, the following complications were found: no complications in 485 (85.8%), complications ≤ IIIa in 59 (10.4%), and severe complications ≥ IIIb in 21 (3.7%) patients. Of the patients, 346 (61.2%) showed improvement; 51 (9.0%), no improvement; and 9 (1.6%), worsening. The grouped outcome (resection weight) is shown in Table 1.

**Table 1:** Grouped short-time outcome distribution (four groups). There were no significant correlations here

			No improvement	Improvement	Worsening	Total
Weight	Group 1 10 to 59.9 g	Number	45	247	7	299
		%	15.1%	82.6%	2.3%	100.0%
	Group 2 60 to 79.9 g	Number	5	46	2	53
		%	9.4%	86.8%	3.8%	100%
	Group 3 80 to 99.9 g	Number	0	17	0	17
		%	0.0%	100.0%	0.0%	100.0%
	Group 4 ≥ 100 g	Number	0	16	0	16
		%	0.0%	100.0%	0.0%	100.0%
Total		Number	50	326	9	385
		%	13.0%	84.7%	2.3%	100.0%

***Influence of surgeon’s experience and resection weight on resection speed:*** The resection speed in the three groups divided according to surgeon’s experience was as follows: 0.9 ± 0.3 (range = 0.1–2.8, median = 0.9; *n* = 246) in group 1, 0.6 ± 0.3 (range = 0.1–2.3, median = 0.6; *n* = 171) in group 2, and 0.4 ± 0.2 (range = 0.1–1.7, median = 0.4; *n* = 143) in group 3. The significance level of the difference between groups 3 and 2, groups 3 and 1, and groups 2 and 3 was high (*p* = 0.001) (Table 2).

**Table 2:** Distribution of the resection speed in the three groups. A significant difference between the surgeons was found in the Kruskal–Wallis test of independent samples. The pairwise comparisons (asymptotic significance: two-tailed tests, significance level of 0.05) proved that all groups differed from each other in pairs

		Resection speed (g/min)	Valid [n]
Experience level	Very experienced	0.9 ± 0.3 (0.1–2.8. Median 0.9)	246
	Experienced	0.6 ± 0.3 (0.1–2.3. Median 0.6)	171
	Little experienced	0.4 ± 0.2 (0.1–1.7. Median 0.4)	143

Significances: Little experience versus experienced: p = 0.001; Little experience versus very experienced: P = 0.001; Experienced versus very experienced: P = 0.001

A two-factor ANOVA was performed to assess the influence of surgeon’s experience and resection weight on the resection speed. With this method, both factors and their interactions could be tested for statistical significance. The analysis revealed that surgeon’s experience (*p* < 0.001), resection weight (*p* < 0.001), and the interaction between them (*p* < 0.01) greatly influenced the resection speed.

With regard to the distribution of Hb loss, interestingly, there were no significant differences between the groups divided according to surgeon’s experience in the Kruskal–Wallis test.

**Significances:** Little experience versus experienced: *p* = 0.001; little experience versus very experienced: *p* = 0.001; experienced versus very experienced: *p*= 0.001.

***Group analysis according to resection weight:*** The patients were divided into groups according to resection weight based on the limits recommended by the EAU guidelines:

To enable a precise differentiation of the risk profile of the resection weights of up to 80 g, we calculated the cut-off values for the occurrence of severe complications ≥ IIIb from the pre- analysis incorporating all resection weights below 80 g using the ROC curve analysis: The ***cut-off values of 59.5 g for the resection weight and 82.5 min for the surgical duration*** could be fixed using the Youden index.

Table 3 shows the distribution of the resection speed in the four groups.

**Table 3:** Distribution of the resection speeds (four groups)

		Resection speed	Valid [n]
Weight	Group 1 10 to 59.9 g	0.6 ± 0.3 (0.1–2.3. Median 0.6)	419
	Group 2 60 to 79.9 g	1.0 ± 0.3 (0.6–2.8. Median 0.9)	71
	Group 3 80 to 99.9 g	1.1 ± 0.4 (0.8–2.6. Median 1.0)	24
	Group 4 ≥ 100 g	1.3 ± 0.3 (0.8–2.3. Median 1.2)	24

The Kruskal–Wallis test showed significant mean differences (p < 0.001). The pairwise comparisons of each group indicated that all groups differed significantly from each other, except for the comparison of group 3 with group 4

### HB loss, administration of blood transfusion, sodium loss, and complications (Clavien-Dindo)

Table 4 shows the distribution of Hb loss.

**Table 4:** Distribution of Hb loss. The Kruskal–Wallis test shows significant mean differences (p < 0.001). All groups differed from each other in pairs, except for groups 3 and 4

		Hb loss (g/dl)	Valid [n]
Weight	Group 1 10 to 59.9 g	1.4 ± 0.9 (0.0–5.1. median 1.3)	387
	Group 2 60 to 79.9 g	2.2 ± 1.3 (0.3–6.9. median 1.9)	73
	Group 3 80 to 99.9 g	2.8 ± 1.8 (1.1–7.7. median 2.2)	24
	Group 4 ≥ 100 g	3.3 ± 1.9 (0.9–8.5. median 2.9)	24

Table 5 shows the distribution of *blood transfusions*. Significant differences (p < 0.001) were found only in group 4 (printed in bold).

**Table 5:** Administration of erythrocyte concentrates (blood transfusion) distribution. Significant differences (p < 0.001) were only found in group 4 (printed in bold)

			Erythrocyte concentrate		Total
			No	Yes	
Weight	Group 1 10 to 59.9 g	Number	404	13	417
		%	96.9%	3.1%	100.0%
	Group 2 60 to 79.9 g	Number	69	4	73
		%	94.5%	5.5%	100%
	Group 3 80 to 99.9 g	Number	23	1	24
		%	95.8%	4.2%	100.0%
	Group 4 ≥ 100 g	Number	17	**7**	24
		%	70.8%	**29.2%**	100.0%
Total		Number	513	25	538
		%	95.4%	4.6%	100.0%

Table 6 shows the grouped distribution of the postoperative sodium level ​​in the four groups. A significant inhomogeneity was detected only in group 4 (printed in bold).

**Table 6:** Postoperative sodium value distribution. Fisher’s exact test shows a p-value less than 0.001. The locus for this significant inhomogeneity is in group 4 (bold print)

			Sodium (mmol/l)			Total
			≤ 120	120—135	≥ 135	
Weight	Group 1 10 to 59.9 g	Number	0	112	304	416
		%	0.0%	26.9%	73.1%	100.0%
	Group 2 60 to 79.9 g	Number	1	22	50	73
		%	1.4%	30.1%	68.5%	100.0%
	Group 3 80 to 99.9 g	Number	0	12	12	24
		%	0.0%	50.0%	50.0%	100.0%
	Group 4 ≥ 100 g	Number	**2**	9	13	24
		%	**8.3%**	37.5%	54.2%	100.0%
Total		Number	3	155	379	537
		%	0.6%	28.9%	70.6%	100.0%

Table 7 shows the grouped distribution of the complications (Clavien–Dindo) in the four groups. The distribution of complications was not homogeneous. The group with resection weights from 60 to 79.9 g had a significantly higher incidence of severe complications ≥ IIIb than the group with resection weights below 60 g (printed in bold).

**Table 7:** Complication distribution

Complications			None	CD ≤ IIIa	CD ≥ IIIb	Total
Weight	Group 1 10 to 59.9 g	Number	369	42	8	419
		%	88.1%	10%	1.9%	100.0%
	Group 2 60 to 79.9 g	Number	57	10	**6**	73
		%	78.1%	13.7%	**8.2%**	100%
	Group 3 80 to 99.9 g	Number	21	0	**3**	24
		%	87.5%	0.0%	**12.5%**	100.0%
	Group 4 ≥ 100 g	Number	14	**6**	**4**	24
		%	58.3%	**25.0%**	**16.7%**	100.0%
Total		Number	461	58	21	540
		%	85.4%	10.7%	3.9%	100.0%

Fisher's exact test showed (p < 0.001) that the distribution of complications was not homogeneous. The group with resection weights from 60 to 79.9 g had a significantly higher incidence of severe complications in comparison to the group with resection weights below 59.9 g. CD = Clavien-Dindo

## Discussion

This work was a retrospective analysis of data collected from patient files. The prostate size was based on the accurate resection weight (what seems to us to be more critical) and not the sonographically estimated preoperative volume.

The main results of this study can be interpreted as follows:

- Increasing surgeon’s experience and resection weight accelerated the resection speed during M-TURP. Significant differences between the individual groups were found with heavier resection weights up to 100 g, leading to a higher resection speed. Despite the further increase in the mean value from 1.1 to 1.3 g/min, the differences in the resection speed between group 4 (over 100 g) and group 3 (80–99.9 g) showed no significance (no further increase > 80 g). We were able to show that the relationship between the duration of the surgery and the amount of tissue resected was not linear. The reason for this finding may be the increased and deeper resection of larger tissue chips at the beginning of M-TURP.
- The cut-off values for the occurrence of severe complications (Clavien–Dindo ≥ IIIb) were 59.5 g for the resection weight and 82.5 min for the surgical time. Hb loss increased proportionally to the resection weight. Significantly more blood transfusion had to be administered in the group with the heaviest resection weight (≥ 100 g). Sodium loss revealed critically low postoperative levels only in the group with the heaviest resection weight.
- The short-term outcome of M-TURP did not significantly differ between the groups.

There are only a few comparable studies in the literature.

Persu et al. achieved an average resection speed of 1.8 mL/min in their study, with 42 of their 100 resections with a preoperative size of > 80 mL being operated on in two stages [11]. The lack of information on the resected prostate tissue, rate of transfusion, and incidence of TUR syndrome prevented a more extensive comparison.

Kwon et al. conducted a study among 48 patients and reported that 19 patients had a mean preoperative prostate size of 124.6 mL [12]. However, only an average of 32.6 mL was resected, and the mean resection speed was 0.28 mL/min.

Marsh and Whitaker achieved an average resection speed of 1.01 g/min in their 102 patients [13]. All patients had a resection weight of > 40 g, and 13 had a resection size of > 80 g. However, with 77 patients who required a transfusion with an average of 1.9 erythrocyte concentrates and 2 patients who died, their values are well above our values.

Herein, the data collected and analyzed in Table 5 on the need to administer blood transfusion were noteworthy. Although there was significant heterogeneity between the groups, this could be explained only by the more than randomly frequent need for the substitution of erythrocyte concentrates in the group with a resection weight of > 100 g (29.2%). Our evaluation led to the conclusion that with an increase in the resection weight, the occurrence of perioperative Hb loss also increases (Table 4), but this is valuable only from a resection weight of > 100 g wherein the administration of erythrocyte concentrates becomes necessary (Table 5).

The probability of TUR syndrome increases with decreasing postoperative serum sodium levels. Table 6 shows that in three patients, the postoperative serum sodium levels dropped below 120 mmol/L, while one patient from the group II had a sodium level of 120 mmol/L. In our evaluation, only the number of severe sodium losses in the group with a resection weight of > 100 g achieved statistical significance.

As indicated in the analyses of Hb and sodium losses, the distribution of complications was also significantly heterogeneous. Table 7 shows how the frequency of serious complications ≥ IIIb increased with an increasing resection weight from 1.9% to 16.7%. This is reflected in the distribution of TUR syndrome: four of the five TUR syndrome cases that occurred in the groups with a resection weight of > 80 g. However, one patient in the group with a resection weight of 80 − 99.9 g developed symptoms of TUR syndrome even with a postoperative sodium level of 127 mmol/L.

In the group with a resection weight of 59.9 g, there was no occurrence of TUR syndrome. In the group with smaller prostate sizes of 10–59.9 g, five of the eight major complications (Table 7) occurred among the patients operated on by less-experienced surgeons. In only 385 patients, the information documented in their files was sufficient to determine the postoperative outcome. Deterioration was noted in 2.3% of the patients; improvement in 84.7%.

As expected, the speed of the resection increased with the expertise and experience of the surgeon (Table 2). Since a surgeon’s experience is not constant but increases with the frequency of performing the procedure, the wealth of experience at the end of the evaluation period was chosen as the parameter. This can mean a lack of clarity in the border areas (for example, from a less-experienced to an experienced surgeon). In contrast, the quality of a surgeon arguably depends on not only the sheer number of operations but also the individual surgical skill. However, the number of operations at the end of the evaluation period seemed to us the best defined from these considerations.

The present findings agree with those of the survey by Cury et al., who demonstrated an increase in the resection speed with an increase in the experience of the surgeon in their analysis of 77 patients [14]. In their study, the average resection speed was 1.07 g/min, and the maximum resection speed was 2.25 g/min in the group operated on by the most experienced surgeons; these values are comparable to those in our group operated on by very experienced surgeons (0.9 and 2.8 g/min, respectively). However, Cury et al. did not analyze the resection speed as a factor related to the resection weight. Since the present study was a retrospective analysis, there was a systematic bias in the distribution of the resection weight across the groups divided according to surgeon’s experience. Thus, 31 of the 48 patients with a resection weight of ≥ 80 g were operated on by very experienced surgeons (65%), 14 by experienced surgeons (29%), and only 3 by less-experienced surgeons (6%). Therefore, we re-examined the influence of surgeon’s experience and resection weight using a two-factor ANOVA. There was a significant increase in the resection speed in association with both surgeon’s experience and resected tissue volume. There was also a significant association between surgeon’s experience and resected tissue volume.

We were able to determine the value limits for the resection weight and surgical time in terms of severe complications: 59.5 g and 82.5 min, respectively. For minor complications, the limit for the resection weight was 57.5 g only. In 2008, Reich et al. also set a value limit of 60 g for the critical resection weight, without going into detail about the determination of this value and without differentiating between slightly severe and severe complications [2]. With an almost identical patient group overall, we were able to confirm this value limit with our work.

This study has several significant limitations to its interpretability, primarily because it was a retrospective analysis. Owing to insufficient documentation, values, ​​such as the preoperative sonographically measured prostate volume, could not be included in our analysis. This is a main criticism, because it severely limits comparability with other studies. Further, the distribution of the patients into the individual groups according to surgeon’s experience was first not randomized; second, a surgeon’s wealth of experience is not fixed but continually develops. Both points imply a bias. We tried to take this was taken into account when examining the speed of resection using the ANOVA. Finally, the data were derived from only a limited number of surgeons from only one department. All of these points lead to the limitation of generality, and thus, generalization of the results should be undertaken with caution.

In the literature, the size of the prostate is usually given as a preoperative sonographically determined value, which, in contrast to the resection weight, can be subject to greater fluctuations and measurement inaccuracies [15, 16]. Since this preoperative sonographic value was not recorded in the present work, a direct comparison with previous studies is difficult.

The resection weight is usually well below the values ​​measured preoperatively, as can be seen in the work of Kwon et al. (32.6 of 124.6 mL = 26.2%) and Cury et al. (12.4 of 45 g = 27.6%, 13.8 of 46.3 g = 29.8%, and 33.3 of 51 g = 65.3%) in which even the most experienced surgeons resect an average of no more than 65% of the tissue measured preoperatively [12, 14]. At best, the latter would estimate the preoperative sonographically measured critical prostate volume in our series to be about 90 g. Other works report average values ​​between 34.7 and 54% [17, 18].

This finding suggests that some of the glands in our patient population had a resection weight well above the 80 g limit preoperatively. Hakenberg et al. and Antunes et al. showed that the percentage of resected tissue has no influence on the outcome [19, 20]. Park et al. reported the missing influence of the amount of resected tissue relative to the volume of the transitional zone [21]. Milonas et al. showed that 30–35% of the entire prostate should be resected to achieve a very good outcome [22]. Yucel et al. were able to show in their work on resection of the prostate with a size of more than 80 g determined via sonography that there were no serious complications [23]. However, the resection weight averaged 52.21 ± 7.59 g and was therefore close to the cut-off value determined herein.

Considering limitations in the interpretability of this purely retrospective study with consequently unmeasurable parameters, as well as the mentioned biases and limited generality, we can cautiously draw some conclusions: Larger prostates are resected with higher speed, but a resection weight of more than 60 g might increase the risk of severe complications. We showed that the resection speed increased with an increasing resection weight. We also confirmed the influence of the experience of the surgeon on the speed of resection.

The indication for M-TURP in patients with a sonographically estimated prostate volume of about 80–90 mL should at least be regarded critically. However, one must also consider individual patient factors and ongoing rapid advances in surgical techniques that could mitigate the risks associated with larger resections.

## Data Availability

No datasets were generated or analysed during the current study.
